# Effects of Copper(II) Oxide on the Co-Pyrolysis of Waste Polyester Enameled Wires and Poly(vinyl chloride)

**DOI:** 10.3390/polym16010027

**Published:** 2023-12-20

**Authors:** Xiaolu Wang, Bingyi Li, Zhidong Xia, Wei Zhou, Yufeng Wu, Zhaoxi Zhu, Guangze Zhu

**Affiliations:** Faculty of Materials and Manufacturing, Beijing University of Technology, Beijing 100124, China; 23B936162@stu.hit.edu.cn (B.L.); zhouwei@bjut.edu.cn (W.Z.); zhuzx@emails.bjut.edu.cn (Z.Z.); zhuguangzebjut@163.com (G.Z.)

**Keywords:** polyester enameled wire, polyvinyl chloride, co-pyrolysis, dechlorination, copper(II) oxide

## Abstract

The emission of chlorinated pollutants is one of the main problems when recovering copper (Cu) via pyrolysis from waste enameled wires. This is mainly attributed to other wastes which possess high poly(vinyl chloride) content, such as electrical wires and cables, which are often recycled together with enameled copper wires. In this research, to control the chlorinated pollutants, copper(II) oxide (CuO) was chosen and demonstrated to be an efficient dechlorinating agent, and CuO did not introduce any impurities that influence the quality of the recovered Cu. The pyrolysis and co-pyrolysis of polyester enameled wires, PVC, and CuO were investigated, and special attention was paid to chlorinated compounds in released pyrolytic products. In particular, the co-pyrolysis of this ternary mixture was studied for the first time, and some new pyrolysis behaviors were discovered. For example, the results of Py-GC/MS analyses showed that the addition of CuO removed about 75% of the chloro-organic products, the main types of which were chloroaromatic compounds rather than the more toxic chloroesters. Moreover, pyrolysis gases were collected and characterized via ion chromatography, and the results showed that the chlorine content in the pyrolysis gases decreased by about 71%. TG analysis indicated that CuO only minimally affected the pyrolysis of polyester paint. However, through the chlorine fixation effect, CuO influenced the dechlorination and dehydrochlorination of PVC, as well as secondary reactions between HCl and pyrolysis products of polyester paint, therefore changing the products and behaviors of co-pyrolysis. Mechanism of reducing chlorine-containing pollutants and reaction mechanism of forming typical pyrolysis products closely correlated to the effects of CuO were also proposed, providing theoretical guidance for the recycling of waste enameled wires.

## 1. Introduction

Thermal processing via pyrolysis is widely used to solve problems involved with plastic waste [1]. For example, pyrolysis is demonstrated to be a promising technology for recovering copper via removing the polymer paint of waste enameled wires [2]. The recycled enameled wires used for copper recovery are generally mixed with wires, cables, and other wastes containing poly(vinyl chloride) (PVC). Therefore, the pyrolysis of waste enameled wires such as polyester enameled wires unavoidably releases chlorinated pollutants due to co-pyrolysis with PVC [3]. Moreover, secondary reactions among pyrolysis products lead to more complex chlorinated pyrolysis products. For instance, secondary reactions with HCl released from PVC pyrolysis produce large amounts of chlorinated organic compounds [4,5,6,7,8]. To reduce the emission of chlorinated pollutants, additives, especially those effective in the dechlorination of PVC, are expected to be useful.

The pyrolysis recovery of PVC has been widely studied, and it is well-known that HCl is the main chlorinated product directly generated from the dehydrochlorination of PVC. Various agents for stabilizing HCl have been developed. For example, alkaline additives including CaCO_3_, Ca(OH)_2_, Na_2_CO_3_, NaOH, etc. act as adsorbents of HCl to immobilize it [9,10,11,12,13,14,15]. Metal oxides such as CuO, ZnO, Fe_2_O_3_, Al_2_O_3_, TiO_2_, V_2_O_5_, MoO_3_, and MnO_2_ chemically react with HCl and produce corresponding metal chlorides [16,17,18,19,20]. However, when recovering Cu via pyrolyzing enameled wires, both alkaline and other metal oxide additives introduce new impurities to the ecovered Cu. Therefore, CuO seems to be a good candidate for dechlorinating waste Cu-enameled wires polluted by PVC. 

The emissions of total chlorine are reduced when PVC is pyrolyzed in the presence of CuO, as reported in the literature [16,21,22]. For example, the total chlorine emissions have been reduced by 40% via thermal degradation in air and 20% via pyrolysis in nitrogen [21]. Additionally, a reduction in chlorine emissions was shown to be dependent on the ratio of PVC to CuO. When the mass ratio was 1:3, only 6.2% of the total chlorine was released in the forms of HCl and Cl_2_ [21]. The formation of polychlorinated dibenzo-*p*-dioxins and dibenzofurans (PCDD/Fs) decreased with the decrease in the molar ratio of PVC/CuO [23]. Besides, CuO was also efficient in decreasing the production of aromatic emissions such as benzene [16,18,21,22]. 

In the pyrolysis process of PVC-CuO, in addition to the decomposition of PVC, reactions between CuO and HCl and reactions between CuO and PVC were supposedly involved [16,24]. CuO reacts with HCl and produces CuCl_2_, which is easily understood and contributes the most to the reduction in HCl emissions. Reacting CuO with PVC was proposed as a solid–solid reaction happening at the beginning of the pyrolysis process [24]. It occurs through binding chlorine via a copper ion and weakening the C–Cl bond of PVC. This is dechlorination instead of dehydrochlorination. Therefore, CuO was intended to accelerate the decomposition of PVC but inhibit HCl emissions. Meanwhile, the PVC crosslinking process was accelerated, which was beneficial to decreasing the yields of aromatic hydrocarbons [22]. 

Although much research on the application of CuO in PVC recycling is available, to the best of our knowledge, there is no research on exploring the use of CuO in waste enameled wire recycling. One study reported that CuCl_2_ acted as a catalyst to promote polyester pyrolysis [25]. This might be another advantage of using CuO since CuCl_2_ should be a reaction product during pyrolysis with the presence of PVC. 

In this research, to reduce chlorinated pollutants in the pyrolysis of polyester enameled wires and PVC, CuO was employed as the dechlorinating agent; its potential can be deduced in part from its effect in PVC recycling, and it would also not introduce any impurities to the system. TG analysis of polyester paint of enameled wires (EPET for short) in the presence of CuO was performed to investigate if CuO has a possible negative effect on EPET pyrolysis. Most importantly, Py-GC/MS of EPET, PVC, and CuO was performed, and the results were analyzed in detail to investigate the pyrolysis products, particularly the chlorinated organic compounds. Moreover, gaseous decomposition products (e.g., HCl) created when pyrolyzing EPET and PVC alone or together with CuO were collected separately and then analyzed via ion chromatography to measure the chlorine contents in the pyrolysis gases. Meanwhile, the chlorine contents in solid residues after pyrolysis were also determined via the Eschka method. Based on the abovementioned, the detailed effects and possible mechanisms of CuO on controlling the emission of chlorine-containing pollutants in the pyrolysis of waste polyester enameled wires were obtained.

## 2. Materials and Methods

### 2.1. Materials

Polyester enameled wires were acquired from Lu’an Yuhua Electrical Co., Ltd., (Lu’an, China). The polyester enameled wire is a non-straight welding enameled wire with a temperature resistance of 130 °C and a diameter of 1.25 mm. PVC was purchased from Shanghai Maclin Biochemical Technology Co., Ltd. The molecular weight of the PVC is between 78 kDa and 84 kDa. Copper(II) oxide (CuO) was acquired from Shanghai Maclin Biochemical Technology Co., Ltd., (Shanghai, China). The surface coating of the polyester enameled wires was stripped off using an electric enameled wire scraper (DF-8), and the stripped polyester paint (EPET) powder with a copper content between 50% and 55% was obtained and used for pyrolysis.

### 2.2. Characterizations

#### 2.2.1. TG Analysis

TG analysis was performed using a TG analyzer (Rigaku TG-DTA 8122, Tokyo, Japan). Three different samples were prepared for TG analysis, including blended EPET and CuO with a mass ratio of 3:1 (3EPET-1CuO for short), 1PVC-3CuO (mass ratio 1:3), and 9EPET-1PVC-3CuO (mass ratio 9:1:3) to determine the (D) TG curves. The sample was thoroughly ground and mixed using agate mortar. Each time sample of 10.0 ± 0.3 mg was placed in an Al_2_O_3_ crucible for testing, and the temperature was set from 30 ℃ to 800 ℃ at a heating rate of 10 ℃/min. The used N_2_ atmosphere had a flow rate of 50 mL/min.

#### 2.2.2. Py-GC/MS Analysis

A pyrolyzer (EGA/PY-3030D, Shimadzu, Japan) coupled with a gas chromatography/mass spectrometry device (GC/MS- QP2010Ultra, Frontier Lab, Koriyama, Japan) was used to perform rapid pyrolysis and product analysis experiments. The pyrolyzer temperatures were set at 440 °C and 640 °C, respectively, and upon pyrolyzing the instrument was preheated to these specified temperatures. The 9EPET-1PVC-3CuO sample was placed in the pyrolyzer and cracked for 12 s. The chromatographic separation of volatile vapor was conducted using an Agilent UA-5MS Capillary Column (30 m × 0.25 mm × 0.25 mm, Agilent, Santa Clara, CA, USA). The GC column oven was heated from 40 °C to 80 °C at a 5 °C/min rate and then to 300 °C at a heating rate of 15 °C/min. The temperature of the GC injection port was 320 °C and the split ratio was 30. The mass spectrometry (MS) analyses were conducted in full scanning mode with an electron energy of 70 eV, ion source temperature of 230 °C, transmission line temperature of 300 °C, and mass scanning range m/z from 40 to 550. High-purity He (99.999%) was used as the carrier gas for GC/MS with a constant flow rate of 1 mL/ min.

#### 2.2.3. Ion Chromatography Analysis

**Figure 1:**
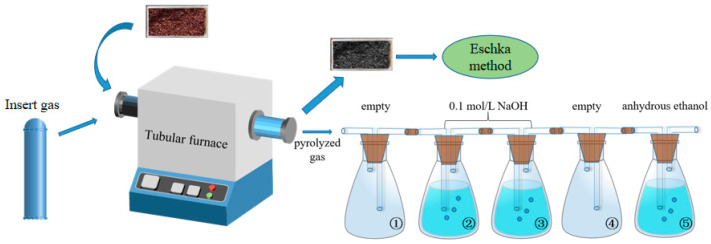
The schematic of the pyrolysis and gas collection units. See Section 2.2.4 for a description of the Eschka method [26,27].

For the PVC, 1PVC-3CuO, 9EPET-1PVC, and 9EPET-1PVC-3CuO samples, their pyrolysis gases were separately collected and then ion chromatography was performed to analyze the chlorine contents. The pyrolysis process was carried out in a tubular furnace (OTF-1200X-60, Siyang Precision Equipment Co., Ltd., Shanghai, China) and the pyrolysis temperature was controlled by a program controller. The schematic of the pyrolysis and collection device is shown in Figure 1. The sample was put into an Al_2_O_3_ crucible and transferred to a tubular furnace. Nitrogen was pre-injected for 10 minutes at a rate of 0.5 L/min and then adjusted to 0.1 L/min for pyrolysis. The collection device consists of five bottle units. The empty bottle ① was set to cool and collect the pyrolyzed oil and prevent reverse suction. Bottles ② and ③ were filled with 250 ml 0.1mol/L NaOH solution and were capable of collecting the chlorine-containing gases. Bottle ④ was empty to prevent reverse suction. Bottle ⑤ with anhydrous ethanol was used to absorb exhaust gas. 

After the pyrolysis process finished, the solutions in bottles ② and ③ were filtered to separate particulates and oily compounds. It was necessary to ensure that the analytic samples were not interfered with by other anions. The filtered liquids from bottles ② and ③ were mixed and stirred for 15 min using a magnetic stirring device, and 10 ml of the liquid was taken for ion chromatography analysis. The ion chromatographic eluent was a mixture of 1.8 mM NaHCO_3_ and 1.7 mM Na_2_CO_3_ in ultra-pure water. For the analysis, chlorine contents in standard NaCl solutions were calibrated. The peak areas of chloride ions in standard solutions with concentrations of 0.00005, 0.00025, 0.0005, 0.0025, and 0.005 mol/L were obtained according to the peak height of chloride ions in the chromatograms, as summarized in Table 1.

**Figure 2:**
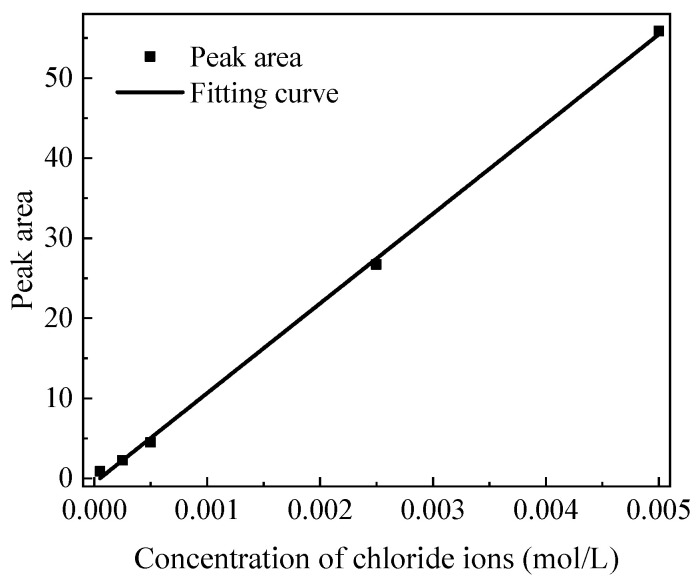
Peak areas vs. concentrations of chloride ions in the standard solutions and the corresponding linear regression fitting curve.

A linear regression line of chloride ion concentration and peak area was fitted as shown in Figure 2, with its regression equation shown in Equation (1), and the fitting correlation coefficient was up to 0.9989, implying a high correlation and high confidence of the fitted curve.

(1) y=(11198 ± 181)c −(0.545 ± 0.455)

where c is the chloride ion concentration and y is the peak area.

#### 2.2.4. Eschka Method

The chlorine contents in the solid residuals after pyrolysis of 1PVC-3CuO, 9EPET-1PVC, and 9EPET-1PVC-3CuO were separately determined via the Eschka method [26,27]. The residuals were ground and mixed with the Eschka mixture. The Eschka mixture is a mixture of magnesium oxide and anhydrous sodium carbonate reagent with a mass ratio of 2:1. The sample was then heated in a muffle furnace to convert the chlorine to chloride. The chloride was leached with boiling water. An excess of silver nitrate solution was added to the acidic medium. With ferric amine sulfate as an indicator, potassium bisulfate was titrated and the amount of chlorine in the solid residual was calculated with the actual consumption of silver nitrate solution. In the titration, one drop of solution was 0.03 ml and the detection limit was set at 0.0003%.

## 3. Results

### 3.1. TG and DTG Results

**Figure 3:**
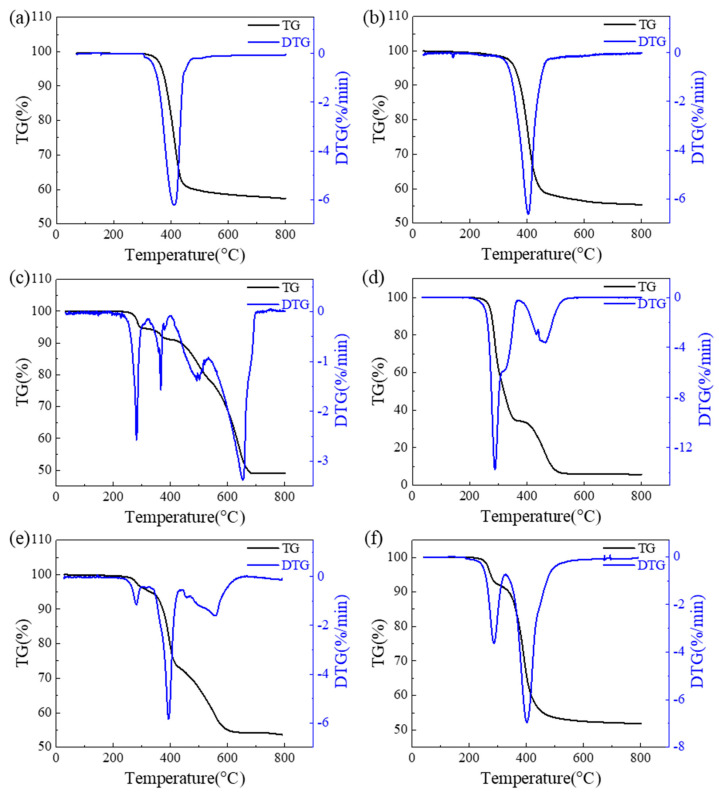
TG and DTG curves of (**a**) 3EPET-1CuO, (**b**) EPET, (**c**) 1PVC-3CuO, (**d**) PVC, (**e**) 9EPET-1PVC-3CuO, and (**f**) 9EPET-1PVC.

The effect of CuO on the thermal degradation behavior of EPET was studied by TG, as shown in Figure 3a,b. The characteristic temperatures in the TG and DTG curves are summarized in Table 2. The pyrolysis process of 3EPET-1CuO had one stage starting from 292 °C to 482 °C. In addition, the peak thermal degradation rate was 6.2%/min and occurred at 409 °C. These characteristics are quite similar to that of EPET [3]. EPET had a single pyrolysis stage ranging from 289 °C to 480 °C with a maximum weight loss rate occurring at 405 °C. The difference between these characteristic temperatures was only within ±4 °C, which indicates that the introduction of CuO to the pyrolysis of EPET did not heavily influence the thermal degradation behavior of EPET.

PVC-3CuO decomposition during pyrolysis characteristically has four stages, as shown in Figure 3c. During the first two stages (238–313 °C, 313–401 °C), it lost 5.5% and 3.5% (totaling 9.0%) of its weight, the third stage was 401–540 °C with a weight loss of 13.2%, and in the fourth stage (540–692 °C) it lost 29.0%. For PVC, as shown in Figure 3d, there were two pyrolysis stages (203–371 °C, 371–536 °C), and the weight loss percentages were 65.3% and 28.9%, respectively. Mathematically, if CuO does not influence the weight loss of PVC, for the PVC-3CuO sample (weight ratio 1:3), its weight loss should be 25% of that of pure PVC. However, when pyrolysis was conducted together with CuO, the sum of the weight losses in the first two stages (238–401 °C) was only 13.8% of the weight loss in the first stage (203–371 °C) of PVC pyrolysis. However, the weight loss in the third stage (401–540 °C) was 45.7% of that in the second stage (371–536 °C) of PVC pyrolysis, which indicates the later increased thermal degradation of PVC. Meanwhile, an additional, fourth stage at a higher temperature range (540–692 °C) appeared in the thermal degradation of PVC-3CuO. 

The thermal degradation of 9EPET-1PVC with and without CuO was further studied. According to Figure 3e,f and Table 2, 9EPET-1PVC-3CuO had three pyrolysis stages of 242–328 °C, 328–439 °C, and 439–636 °C with weight loss percentages of 4.3%, 22.5%, and 18.3%, respectively, while 9EPET-1PVC had two stages of 214–325 °C and 325–528 °C with weight loss percentages of 8.5% and 39.5%, respectively. Similar to the analysis outlined in the previous paragraph, if ignoring the effect of CuO and according to the mass ratios, the mathematically calculated weight loss of 9EPET-1PVC-3CuO should be 76.9% of that of 9EPET-1PVC. However, comparing the first pyrolysis stages of the two samples, the proportion was only 50.6%. Meanwhile, although the proportion of weight loss in stage II of EPET-PVC-CuO pyrolysis accounted for less than the mathematically calculated one, it should be noted that the upper limit of this temperature range (328–439 °C) is 89 °C lower than that (325–528 °C) of EPET-PVC. Additionally, EPET-PVC-CuO also has an additional pyrolysis stage at a higher temperature range (439–636 °C). We think this might be attributed to the delayed decomposition of polymers, especially PVC, in the presence of CuO; additionally, according to the literature [16], in the latter pyrolysis process, carbon atoms in char react with chloride salts and generate volatile products. 

There are several similar changing trends between the two above-discussed pairs of samples, 1PVC-3CuO/PVC and 9EPET-1PVC-3CuO/9EPET-1PVC, when comparing the characteristic parameters of the two samples of each pair. The presence of CuO led to much lower weight losses in the early pyrolysis stages, and the beginning temperatures of weight losses had 35 °C and 28 °C delays, respectively. These demonstrated that CuO decreases and adversely affects the emission of the volatile pyrolysis products produced. However, all of the peak DTG temperatures in the first pyrolysis stage of the 1PVC-3CuO, 9EPET-1PVC-3CuO, and 9EPET-1PVC samples, which were 281, 279, and 284 °C, were unaffected by EPET and were very comparable to that of pure PVC (288 °C). Additionally, CuO rarely influences EPET pyrolysis; additionally, as demonstrated in the literature, most of the weight loss of 9EPET-1PVC in early pyrolysis results from PVC decomposition [28]. The first stage of PVC pyrolysis is very important for studying the emissions of chlorine-containing pollutants, as it is well known that most of the chlorine is released in the form of HCl in the first pyrolysis stage and contributes the most to weight loss [5,7,16]. Since the temperature ranges of the first and second pyrolysis stages of 9EPET-1PVC-3CuO overlapped with that of the first stage of PVC pyrolysis, it can be deduced that the changing trends in these two stages are closely related to the interactions between CuO and PVC, and it is reasonable to estimate that CuO inhibits HCl emission and/or stabilizs HCl into a solid, resulting in decreased weight loss in the early co-pyrolysis stages of EPET, PVC, and CuO. 

### 3.2. Py-GC/MS Results

In order to further study the co-pyrolysis of EPET and PVC in the presence of CuO, Py-GC/MS was performed on the 9EPET-1PVC-1CuO sample to analyze the main organic pyrolysis products. The first detection temperature was set at an integer value of 440 °C which is approximately equal to the termination temperature of the second pyrolysis stage (439 °C). This setting was chosen because, as discussed previously, in the first two pyrolysis stages, the interactions between CuO and PVC are significant, and the pyrolysis products can reflect these interactions. The second detection temperature was set at 640 °C, which is after the end of the whole pyrolysis process, to detect the final pyrolysis products. Table 3 summarizes the main pyrolysis products with contents larger than 1wt.%. At 440 °C, the pyrolysis products mainly consisted of benzoic acid, 1,2-ethyleneglycol dibenzoate, propylene ketone, and vinyl formate, and the total content accounted for 73.85 wt.% of the products. These products were demonstrated to be mostly derived from EPET [3,8,29]. Benzene was also detected in the products, though in a small amount (2.24 wt.%). Benzene is one of our main concerns since it is a main product of PVC in early pyrolysis stages and is strongly correlated with other accompanying products such as HCl, etc. In our previous study [3] where CuO was absent, the 9EPET-1PVC pyrolysis produced 13.21wt.% of benzene at a temperature of 325 °C, and until this temperature, the weight loss of this sample was much lower than that of 9EPET-1PVC-1CuO at 439 °C (8.5% vs. 26.8%). This result illustrates that the presence of CuO decreases the yield of benzene, which is also in accordance with the literature [16,18,21,22]. Meanwhile, the reduction in benzene emission indicates the reduced formation of polyenes, as it is the decomposition of polyenes and the following aromatization of produced cyclohexene and cyclohexadiene rings that generate benzene [7]. Several studies have also reported that CuO promotes the crosslinking of PVC and thereafter inhibits the generation of polyenes. Therefore, it can be deduced that in the co-pyrolysis of EPET, PVC, and CuO, the crosslinking of PVC is prominent.

At 640 °C, the main products turned out to be benzoic acid, 1,2-ethyleneglycol dibenzoate, benzene, ethylamine, propylene ketone, vinyl benzoate, vinyl formate, etc. Comparing the pyrolysis products at 440 °C and 640 °C, the contents of benzoic acid, benzene, biphenyl, and vinyl benzoate increased and the contents of other substances decreased. These trends might be because the molecules of pyrolysis products tend to be smaller and the structures become simpler with the increase in the pyrolysis temperature. Additionally, ethylamine appeared in the products, and we believe it derived from EPET since the elemental analysis showed that the EPET contained 0.46 wt.% nitrogen [3]. Meanwhile, the content of benzene increased to 7.39 wt.%, which might not only be attributed to PVC pyrolysis, since at high temperatures the main product, benzoic acid, from EPET can decompose and generate benzene [8,11,30]. Therefore, at 640 °C, the benzene cannot be regarded as a standard to evaluate PVC pyrolysis alone. 

It is particularly noteworthy that no chlorine-containing compound was found in products with contents over 1 wt.% at 440 and 640 °C. Nevertheless, to better understand the pyrolysis mechanism, the main organic chlorinated compounds (COCs) and their contents are summarized in Table 4. The products are characteristic of four main types, including chloroaromatics, chloroaromatic derivatives, chloroesters, and acyl chlorides. At 440 °C, the total COC content was 2.54 wt.%, while at 640 °C it increased to 3.35 wt.%, which indicates that a higher temperature may benefit the formation of COCs. A similar tendency was also observed for the 9EPET-1PVC sample, as reported in our previous publication [3]; namely, at 325 °C, which was the end temperature of the first pyrolysis stage, there was only 0.35 wt.% of COCs, and at end the pyrolysis at 528 °C the content reached 11.6 wt.%. Comparing the two samples, it can be clearly seen that CuO dramatically reduced the emission of COCs in the co-pyrolysis of EPET and PVC. Additionally, the most abundant products changed from chloroesters (9.28 wt.%) to chlorinated aromatics and their derivatives, and the contained chloroesters were only 0.34 wt.%. As demonstrated previously, chloroesters formed from an addition reaction between the HCl and C=C bond of the vinyl esters that were generated in EPET pyrolysis [3,8]. Here, in 9EPET-1PVC-3CuO pyrolysis, this addition reaction was greatly hindered. Since CuO does not affect EPET pyrolysis, it can be deduced that it influences HCl or this addition reaction. 

It was also found that in the chlorine-containing aromatics and their derivatives, chlorine atoms were often directly attached to benzene rings. For example, there was 0.91 wt.% chlorobenzene in the total 1.23 wt.% of the chloroaromatics. It was demonstrated that chlorobenzene is the main chlorinated hydrocarbon from PVC pyrolysis [5,21,31,32]. Additionally, the formation of chlorobenzenes was intended to rechlorinate the polyenes, which was achieved via the recombination of chlorine with double bonds, and Cu (II) was suggested to be able to promote this rechlorination [33]. However, due to decreased polyenes in the co-pyrolysis, the amount of chlorobenzenes was very low. Additionally, another possible mechanism of chlorobenzene formation was proposed to be the Friedel–Crafts chlorination of benzene through Lewis acid, and the literature reports that FeCl_3_ was the Lewis acid [34]. Cu (II) is a weak Lewis acid, and therefore this Friedel–Crafts chlorination may have also occurred in the present pyrolysis system. 

Other typical chorine-containing products with relatively high proportions are chlorobenzoic acids and their derivatives, including 2-chlorobenzoic acid, 2,5-dichlorobenzoic acid, and 2-chlorobenzoic acid-2-oxo-2-phenylethyl ester. Compared with the pyrolysis products of 9EPET-1PVC, among which there was only a very small amount of 3-chlorobenzoic acid-2-chloroethyl ester, herein, the amounts of chlorobenzoic acids and their derivatives increased and their types varied. As benzoic acid was the dominant pyrolysis product in the co-pyrolysis (see Table 3) and was generated from EPET, it is reasonable to assume a chlorination of benzoic acid that produces chlorobenzoic acids. A study on learning the effect of CuCl_2_ on the thermal degradation of PET found that there was also chorobenzoic acid in the products; however, no further explanation was given [29]. According to the abovementioned results, we assume that the generated CuCl_2_ from the reaction of CuO and HCl promotes the formation of chlorobenzoic acids and their derivatives. The reaction mechanism is discussed in Section 3.4.

To clarify the chlorine distribution in liquid pyrolysis products, Table 5 summarizes the fractions of chlorinated organic compounds for the 9EPET-1PVC-3CuO sample pyrolyzed at 640 °C. According to the carbon atom number, the chlorinated liquid products were separated into three categories (C5–C9, C10–C13, and >C13). It can be seen that the chloroaromatics and their derivatives were distributed in all the categories, but the light liquids (C5–C9) were the main fractions. Meanwhile, chloroesters were only found in medium liquids (C10–C13).

### 3.3. Determination of Chlorine Contents in Pyrolysis Gas and Solid Products

#### 3.3.1. Ion Chromatography Analysis

To study the chlorine content in pyrolysis gas products, we collected the pyrolysis gases of PVC, 1PVC-3CuO, 9EPET-1PVC, and 9EPET-1PVC-3CuO, respectively, and then analyzed them via ion chromatography. The peak areas of chloride ions in the collected solutions were obtained. Additionally, the chloride ion concentration c was calculated using Equation (1) and the chloride ion content $w_{t}$ was calculated using Equation (2). The contents of PVC in all samples were kept at 0.1 g.

(2) $$w_{t}=\frac{c\times V\times M}{m}=\frac{c\times 0.5\times 35.45}{0.0567}$$

where $w_{t}$ is the calculated chloride ion content; *c* is the chloride ion concentration in mol/L; *V* is the volume of the collection fluid in L; *M* is the relative atomic mass of chlorine; m is the total mass of chlorine. The data were compiled and the calculated chloride ion contents in the pyrolysis gas of the four samples are shown in Table 6.

Table 6 shows that the highest chlorine content (54 wt.%) in the gas took place during PVC pyrolysis. The chlorine content in the gas products greatly decreased to 14 wt.% when CuO was added to the PVC, which is 40% less compared with that of PVC pyrolysis. This indicates that CuO has a chlorine fixation effect on the HCl released during the pyrolysis of PVC. When EPET was co-pyrolyzed with PVC, the chlorine content decreased to 24 wt.%, which is 30% less than that in PVC pyrolysis. This indicates that EPET reduces the chlorine content of pyrolysis gas in the co-pyrolyzed EPET–PVC system. In previous studies, it was reported that HCl reacted with esters and vinyl esters produced by PET pyrolysis to form chlorinated organics [8,35,36]. In the co-pyrolyzed process of EPET, PVC, and CuO, the chlorine content in the pyrolysis gas decreased to the lowest value, accounting for only 7 wt.% of the total chlorine mass. The decrease in chlorine in the pyrolysis gas should be primarily due to the fixation of HCl in the solid and oil phases, which are reactions of HCl when released from pyrolyzing PVC with CuO to form CuCl_2_ [37], as well as the addition reaction of HCl with vinyl esters produced from EPET pyrolysis, respectively. Meanwhile, this decrease in HCl would eliminate the well-known autocatalysis of PVC dehydrochlorination by HCl, which should be one of the reasons for the delayed thermal degradations of PVC-3CuO and 9EPET-1PVC-3CuO (see Section 3.1).

#### 3.3.2. Eschka Method Analysis

The contents of chlorine in pyrolysis solid residues of 1PVC-3CuO, 9EPET-1PVC, and 9EPET-1PVC-3CuO were studied via the Eschka method, and the chlorine content $W_{cl}$ in the solid was calculated using Equation (3); the detailed experimental parameters and calculated results are shown in Table 7, where $W_{cl}$ is the chlorine content; $V_{1}$ is the measured sample volume; c is the concentration of potassium thiocyanate standard solution in mmol/mL; $M_{cl}$ is the relative atomic mass of chlorine; and *m* is the total mass of chlorine.

(3) $$W_{cl}=\frac{V_{1}\times c\times M_{cl}}{m}\times 100$$

Substituting $W_{cl}$ into Equation (4) yields the chlorine in the solid residues as a percentage of the total chlorine, $w_{Cl}$. The results are shown in Table 8.

(4) $$w_{Cl}=\frac{W_{cl}\times m}{0.0567}\%$$

At the end of pyrolysis, the chlorine contents in the solid residues of 1PVC-3CuO, 9EPET-1PVC, and 9EPET-1PVC-3CuO were 7.39 wt.%, 5.75 wt.%, and 19.7 wt.%, respectively. This illustrates that the 9EPET-1PVC-CuO sample had the best chlorine fixation effect. In other words, the presence of both EPET and CuO improved the chlorine fixation effect the most, followed by CuO and then EPET. 

As summarized in Table 9, compared with the results in the literatures [16,21,33], the reduction in HCl emission in this work is the greatest, while similar to that in [21]. Chloroaromatics are the main COCs in the pyrolysis products, but their total amount was much lower in the present work. From the results of the organic (oil)–gas–solid tri-state analysis of the pyrolysis products, it can be found that, unlike the pyrolysis of PVC alone, when a small amount of PVC was co-pyrolyzed with EPET, chlorine entered the oil phase in large quantities and existed as chlorinated organic compounds while reducing the emission of chlorine in the gaseous products. When CuO was added into the 9EPET-1PVC system, the release of chlorine in the gas was further reduced, and the chlorinated organic compounds in the oil were reduced as well, while chlorine content in the solid residue increased.

### 3.4. Proposed Reaction Mechanism of Co-Pyrolysis

**Scheme 1:**
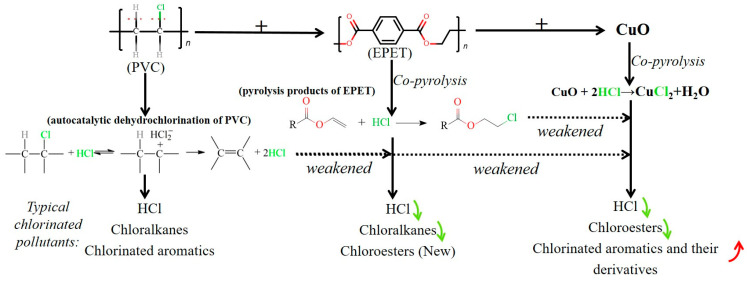
The variation of the main chlorinated pollutants from the pyrolysis of PVC, 9EPET-1PVC, and 9EPET-1PVC-3CuO. The green arrows indicate decrease of content while the red one means increase.

Based on the abovementioned results and discussion, the variation of the main chlorine-containing pollutants from the pyrolysis of PVC, 9EPET-1PVC, and 9EPET-1PVC-3CuO is summarized and shown in Scheme 1. The emission of HCl dramatically decreased in the co-pyrolysis of EPET, PVC, and CuO. Its variation mechanisms are as follows. (i) In PVC pyrolysis, a large amount of HCl was produced, and HCl also acted as a catalyst for the further dehydrochlorination of PVC, as shown on the left of Scheme 1; (ii) in 9EPET-1PVC pyrolysis, the HCl formed from PVC was mainly consumed by an addition reaction with vinyl esters formed from EPET, as shown in the middle of Scheme 1; and (iii) in 9EPET-1PVC-3CuO pyrolysis, besides the addition reaction described in (ii), HCl could also be fixed by CuO in the solid phase by forming CuCl_2_. 

**Scheme 2:**
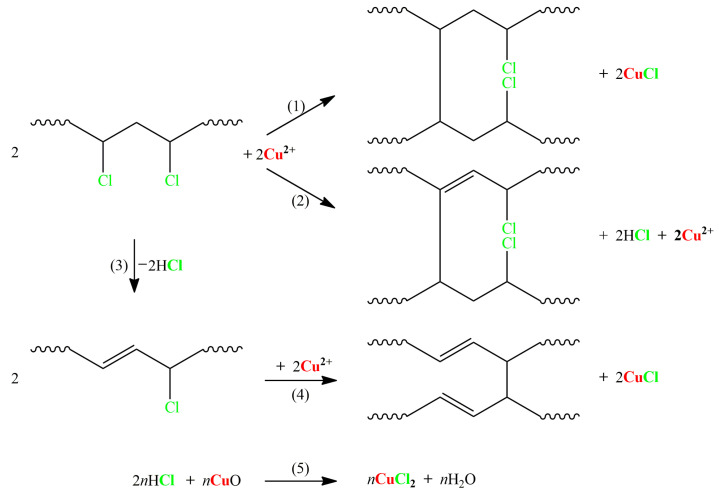
The possible reactions between CuO and PVC in the pyrolysis process.

**Scheme 3:**
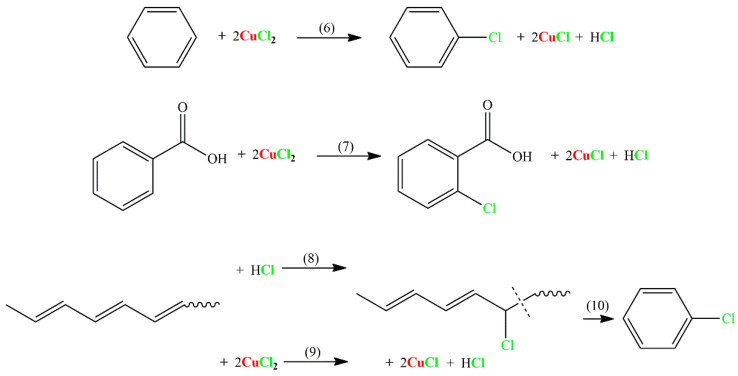
The possible reactions generated from chlorobenzene and chlorobenzoic acid.

Besides this research, there exist several studies that have investigated the effects of Cu (II) on PVC pyrolysis; therefore, there exist plenty of foundations upon which we can systemically summarize the reaction mechanisms. Herein, according to the literature and the results of this research, possible reactions between Cu (II) and PVC as well as chlorinated organic formation reactions closely related to Cu (II) in the co-pyrolysis process are proposed, as shown in Scheme 2 and Scheme 3. Reactions (1), (2), and (4) in Scheme 2 show the crosslinking of PVC itself and its product, polyene, respectively, in the presence of Cu (II) [7,16,38]. Additionally, Reactions (1) and (4) are reductive coupling mechanisms that benefit HCl fixation, while Reaction (2) is the Lewis acid mechanism and produces HCl. However, the produced HCl in Reactions (2) and (3) can be absorbed by CuO, as indicated in Reaction (5). These mechanisms are not only supported by the fact that HCl emissions are reduced, but the reduced benzenes in the pyrolysis products also indicate an enhanced crosslinking of PVC and polyene, as the crosslinking was demonstrated to greatly inhibit the generation of benzenes [16,21,22,33,38].

For chlorinated organics, the addition of CuO causes a dramatic decrease in chloroesters in the product, and this is attributed to decreased HCl formation that leads to a weakened addition of HCl to vinyl esters, as indicated in the middle of Scheme 1. Moreover, the main product type turns out to be chlorinated aromatics and their derivatives, for example, chlorobenzene and chlorobenzoic acid. The possible reactions that generate these products are listed in Scheme 3. Reactions (6) and (7) show a mechanism of Friedel–Crafts chlorination of benzene and benzoic acid through the Lewis acid CuCl_2_, respectively [34,39,40]. Reactions (8–10) represent the recombination of chlorine with double bonds of polyene followed by cyclization reactions (10), resulting in chlorobenzene [5,34,41]. Particularly, it was reported that Lewis acids such as CuCl_2_ accelerate the recombination and are beneficial to chlorobenzene generation (Reactions 9–10) [34,41]. It is noteworthy that although the representative reactions shown in Scheme 3 are feasible, they are not dominant secondary reactions during the pyrolysis process because the reaction products (e.g., chlorinated aromatics) only account for a small amount of the pyrolysis products (Section 3.2). It also should be mentioned that although Scheme 1, Scheme 2 and Scheme 3 comprehensively list the main and most likely reaction mechanisms that affect the generation of chlorine-containing pollutants, and the reaction mechanisms were carefully deduced from the results of this research and those in the literature, parts of them still require further study and proof.

## 4. Conclusions

The results of the TG analyses show that CuO affects the thermal degradation behavior of EPET-PVC through delaying the termination temperature from 528 °C to 636 °C and increasing the pyrolysis stages to three. With the addition of CuO to the EPET-PVC pyrolysis system, the contents of the released organic chlorinated compounds and HCl pollutants were reduced by about 75% and 71%, respectively. Additionally, the types of chlorine-containing pollutants and their corresponding contents in the gas, liquid (oil), and solid phases were also redistributed. The main reasons for these results lie in the effects of CuO on PVC pyrolysis. HCl released from the pyrolysis of PVC can be fixed by CuO in the form of CuCl_2_, which reduces HCl emissions and the autocatalytic dehydrochlorination of PVC, resulting in delayed thermal degradations in PVC-containing samples. Meanwhile, due to the action of CuO, the addition reaction of HCl to the double bond of vinyl esters generated via EPET pyrolysis was weakened, leading to a reduction in chloroester content from 9.28% to 0.34% according to Py-GC/MS analyses. CuO promoted the crosslinking of PVC and polyenes, allowing less production of benzenes. The CuCl_2_ generated from the secondary reactions during the pyrolysis process helped to promote the rechlorination of benzenes, benzoic acids, and polyenes, which resulted in the chloroaromatics and their derivatives accounting for 2.41% of the detected organic pyrolysis products and becoming dominant chlorinated organic products. Based on these results, this research provides technical support and theoretical guidance for the clean emission of pyrolysis products of waste enameled wire containing chlorine impurities.

## Figures and Tables

**Table 1:** Chromatographic peak areas of chloride ions in the standard solutions of different concentrations.

Concentration (mol/L)	Peak Area
0.00005	0.884
0.00025	2.249
0.0005	4.508
0.0025	26.724
0.005	55.857

**Table 2:** Characteristic pyrolysis temperatures and weight losses of different samples.

Sample	Temperature Range (°C)/Weight Loss (%)				Temperature of DTG Peak (°C)			
	I	II	III	IV	I	II	III	IV
3EPET-CuO	292–482	-	-	-	409	-	-	-
EPET	289–480	-	-	-	405	-	-	-
PVC-3CuO	238–313/5.5	313–401/3.5	401–540/13.2	540–692/29	281	365	494	653
PVC	203–371/65.3	371–536/28.9	-	-	288	454	-	-
9EPET-1PVC-3CuO	242–328/4.3	328–439/22.5	439–636/18.3	-	279	392	554	-
9EPET-1PVC	214–325/8.5	325–528/39.5	-	-	284	401	-	-

**Table 3:** EPET-PVC-CuO pyrolysis products with contents over 1 wt.%.

Product	Retention Time (min)	Product Content (wt.%)	
		440 °C	640 °C
Ethylamine	1.505	-	4.60
Vinyl formate	1.562	7.19	2.92
2-Propenal	1.703	10.86	3.11
Benzene	2.843	2.24	7.39
Vinyl benzoate	12.868	1.31	3.05
Benzoic acid	14.633	41.89	48.27
Biphenyl	15.627	0.27	1.26
Ethyl benzoate	15.987	1.18	0.05
Diethylene glycol dibenzoate	21.523	1.50	0.63
1,2-Ethanediol dibenzoate	21.502	13.91	10.24

**Table 4:** Chlorinated organic compounds produced in the pyrolysis process of EPET-PVC-CuO.

Product	Retention Time (min)	Product Content (wt.%)	
		440 °C	640 °C
Allyl chloride	1.840	-	0.1
1,6-Dichlorohexane	2.305	-	0.03
**Chloroaromatics** ^1^↓		**0.06 ^†^**	**1.23 ^†^**
Chlorobenzene	5.790	-	0.91
1,4-Dichlorobenzene	10.310	-	0.14
1,2-Dichlorobenzene	11.310	0.01	0.01
1,2,3-Trichlorobenzene	13.400	-	0.06
2,4,6-Trichlorobenzene	15.555	0.05	-
2-Chlorobiphenyl	16.730	-	0.11
**Chloroaromatic derivatives** ^1^↓		**2.1 ^†^**	**1.18 ^†^**
2-Chlorobenzoic acid	17.375	0.54	0.15
2,5-Dichlorobenzoic acid	18.850	0.37	0.02
1-(4-Chlorophenyl)-2-phenylethan-1,2-dione	19.400	0.20	0.13
2-Chloro-2-phenyl acetophenone	22.170	-	0.14
2-Chlorobenzoic acid-2-oxo-2-phenylethyl ester	22.525	0.96	0.62
3,4-Dichloro-benzophenone	23.665	0.03	0.12
**Chloroesters** ^1^↓		**0.34 ^†^**	**0.38 ^†^**
4-Ethylbenzoic acid-2-chloroethyl ester	17.750	0.15	-
Phthalic acid-di(2-chloroethyl) ester	19.505	-	0.38
Isophthalic acid-2-chloroethyl ethyl ester	19.570	0.19	-
**Acyl chlorides** ^1^↓		**0.04 ^†^**	**0.43 ^†^**
p-Chlorobenzoyl chloride	14.830	0.04	0.32
p-Phenylphenacyl chloride	21.910	-	0.11

^1^ Types of products and their corresponding ^†^ total contents.

**Table 5:** The fractions of chlorinated organic compounds in the liquid phase of the 9EPET-1PVC-3CuO sample pyrolyzed at 640 °C.

Fractions	Chlorinated Products	Content (wt.%)
C5–C9	Aromatics	1.29
	Esters	-
	Others	0.35
C10–C13	Aromatics	0.23
	Esters	0.38
	Others	-
>C13	Aromatics	0.89
	Esters	-
	Others	0.11
Total aromatics		2.41
Total esters		0.38

**Table 6:** Chlorine content ($w_{t}$) in pyrolysis gas of different samples.

Sample	Peak Area	$w_{t}\;$(wt.%)
PVC	18.63	54
1PVC-3CuO	8.1	14
9EPET-1PVC	4.92	24
9EPET-1PVC-3CuO	2.09	7

**Table 7:** Parameters for the calculation of chlorine content ($W_{cl}$) in the pyrolytic solid residues of different samples.

Sample	Temperature (°C)	Weighing Mass *m* (g)	Sampling Volume (mL)	Potassium Thiocyanate Concentration *c* (mmol/mL)	Test Sample Volume *V*_1_ (mL)	$W_{cl}\;$(wt.%)
1PVC-3CuO	692	0.2271	250.00	0.02632	4.50	1.85
9EPET-1PVC	528	0.3980	250.00	0.02632	3.50	0.82
9EPET-1PVC-CuO	640	0.6402	250.00	0.02632	12.00	1.75

**Table 8:** The content of chlorine in the solid residues accounting for the total chlorine in the sample.

Sample	Temperature (°C)	$w_{Cl}$ (wt.%)
1PVC-3CuO	692	7.39
9EPET-1PVC	528	5.75
9EPET-1PVC-CuO	640	19.7

**Table 9:** Comparison with studies by other authors.

Authors	Sample	Temperature (°C)	Main Results
Wang et al. [24]	PVC-CuO	190	CuO was chlorinated by PVC at 190 °C.
Ji et al. [18]	PVC-CuO	280	(i) CuO absorbed the HCl produced by pyrolysis; (ii) the production of benzene was significantly reduced.
Meng et al. [16]	PVC-CuO	310	The proportion of HCl decreased from 94.7% (for PVC) to 80.9%.
Blazsό and Jakab [33]	PVC-CuO	550	(i) Two-thirds of the HCl emissions were reduced; (ii) benzene formation was hindered.
Grimes et al. [21]	PVC-CuO	300, 600	(i) Chloroaromatics were the main chlorinated organic compounds (COCs); (ii) chlorine emissions were reduced by 20%; (iii) benzene emissions were reduced by more than 90%.
Wang et al. (this work)	EPET-PVC-CuO	440, 640	(i) Chloroaromatics were the main COCs; (ii) COCs were reduced by 75%; (iii) HCl emissions were reduced by 71%.

## Data Availability

Data are contained within the article.
